# Macular dystrophy in Kabuki syndrome due to *de novo KMT2D* variants: refining the phenotype with multimodal imaging and follow-up over 10 years: insight into pathophysiology

**DOI:** 10.1007/s00417-023-06345-1

**Published:** 2024-01-11

**Authors:** Veronika Vaclavik, Aurelie Navarro, Alain Jacot-Guillarmod, Armand Bottani, Young Joo Sun, Joel A. Franco, Vinit B. Mahajan, Vasily Smirnov, Isabelle Bouvet-Drumare

**Affiliations:** 1 Jules-Gonin Eye Hospital, University of Lausanne, Lausanne, Switzerland; 2 Department of Ophthalmology, Hospital Cantonal, Fribourg, Switzerland; 3 Service of Genetic Medicine, Geneva University Hospitals, Geneva, Switzerland; 4 Molecular Surgery Laboratory, Byers Eye Institute, Stanford University, Palo Alto, CA USA; 5 Veterans Affairs Palo Alto Health Care System, Palo Alto, CA USA; 6 Exploration de la Vision et Neuro-Ophtalmologie, CHU de Lille, Lilles, 59000 France; 7 Univ. Lille, Inserm, CHU Lille, U1172-LilNCog-Lille Neuroscience & Cognition, Lille, F-59000 France

**Keywords:** Kabuki syndrome, Macula, Dystrophy, Retinal imaging, Adaptive optics, Autofluorescence imaging, Multimodal imaging, *KMTD2* gene

## Abstract

**Background:**

Kabuki Syndrome is a rare and genetically heterogenous condition with both ophthalmic and systemic complications and typical facial features. We detail the macular phenotype in two unrelated patients with Kabuki syndrome due to *de novo* nonsense variants in *KMT2D*, one novel. A follow-up of 10 years is reported. Pathogenicity of both *de novo* nonsense variants is analyzed.

**Methods:**

Four eyes of two young patients were studied by full clinical examination, kinetic perimetry, short wavelength autofluorescence, full field (ff) ERGs, and spectral-domain optical coherence tomography (SD-OCT). One patient had adaptive optic (AO) imaging. Whole exome sequencing was performed in both patients.

**Results:**

Both patients had de novo nonsense variants in *KMTD2*. One patient had c.14843C>G; p. (Ser4948ter) novel variant and the second c.11119C>T; p. (Arg3707ter). Both had a stable Snellen visual acuity of 0.2–0.3. The retinal multimodal imaging demonstrated abnormalities at the fovea in both eyes: hyperreflectivity to blue light and a well-delimited gap—disruption of ellipsoid and interdigitation layer on OCT. The dark area on AO imaging is presumed to be absent for, or with structural change to photoreceptors. The ff ERGs and kinetic visual fields were normal. The foveal findings remained stable over several years.

**Conclusion:**

Kabuki syndrome–related maculopathy is a distinct loss of photoreceptors at the fovea as shown by multimodal imaging including, for the first time, AO imaging. This report adds to the literature of only one case with maculopathy with two additional macular dystrophies in patients with Kabuki syndrome. Although underestimated, these cases further raise awareness of the potential impact of retinal manifestations of Kabuki syndrome not only among ophthalmologists but also other healthcare professionals involved in the care of patients with this multisystem disorder.

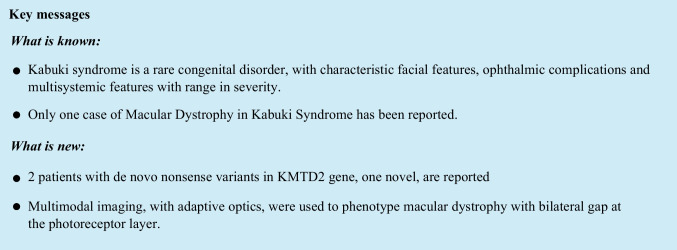



## Introduction

Kabuki syndrome (KS; OMIM 147920) was first described in Japan in 1981 by 2 independent authors, Niikawa [1] and Kuroki J [2]. Initially, the syndrome was specific to the Japanese population, where the estimated prevalence is 1/32,000 [3]. Later, there were reports of KS in all ethnic groups, including European, Brazilian, Chinese, Arabic, Mexican, Chinese, and African [4].

KS is a condition with multiple congenital anomalies and intellectual disabilities. Consensus clinical diagnostic criteria were recently published [5]. The criteria define characteristic features including postnatal growth retardation, developmental delay, intellectual disability, and typical facial features: long palpebral fissures with eversion of the lateral third of the lower lid, depressed nasal tip, arched and broad eyebrows with lateral third being sparse or notched, and large or cupped ears. Additional findings include dermatoglyphic abnormalities (persistent fingertip pads), skeletal anomalies as well as heart, kidney or gastrointestinal abnormalities, microcephaly, or short stature [5–7].

An association between KS and pathogenic variants in *KMT2D* (also known as *MLL2*; OMIM # 602113) was first described in 2010 and defines Kabuki syndrome 1, KS1 [8]. About 60–75% of cases with clinical diagnosis of Kabuki syndrome have a disease-causing variant in *KMT2D*, encoding a histone-lysine *N*-methyltransferase 2D protein [6]. Some patients (3–8%) have pathogenic variants in *KDM6A* (OMIM # 300128), an X-linked gene which encodes lysine specific demethylase 6A and is defined as Kabuki syndrome 2 (KS2; OMIM # 300867).

Although underestimated, the published prevalence of ophthalmological abnormalities in KS is between 38 and 61% [9–11]. The most common are strabismus, ptosis, iris and retinal coloboma, or choroidal coloboma. Blue sclera, cataracts, nystagmus, refractive error, microphthalmia, anophthalmia, optic nerve hypoplasia, micro- or megalo-cornea, as well as corneal staphyloma, and Salzmann nodular degeneration have also been described [10, 12–16].

Specific retinal findings in KS are less known: retinal telangiectasias, macular deposits, and only one case of macular dystrophy have been reported [17–19].

We report two unrelated female patients with macular dystrophies and *de novo* nonsense variants in *KMT2D*: a known c.11119C>T; p.(Arg3707ter) pathogenic variant and a novel c.14843C>G; p.(Ser4948ter) variant. A 10-year long follow-up, as well as multimodal imaging, provides additional insight into this possibly underestimated KS-related macular dystrophy.

## Material and methods

### Clinical assessment

The protocol of the study adhered to the tenets of the Declaration of Helsinki and was approved by CER-VD, (Canton Ethics committee for research on human, Lausanne, Switzerland) Req: 2020-02358.

Both patients have over 10 years of clinical follow-up. Subjects underwent full ophthalmic examination including color fundus photography and autofluorescence (AF) imaging. AF imaging was obtained using scanning laser ophthalmoscope (HRA2, Heidelberg Engineering, Heidelberg, Germany) by illuminating the fundus with argon laser light (488 nm) and viewing the resultant fluorescence through a band-pass filter with a short wavelength cut off at 495 nm. Further tests were performed including Goldmann perimetry and optical coherence tomography (OCT), using the Spectralis HRA2 (Heidelberg Engineering, Heidelberg, Germany). Adaptive optics were recorded in one patient attending Jules Gonin, using an adaptive optics machine from BMC Apaeros (Boston Micromachines Corporation. 30 Spinelli Place, Cambridge, MA, USA).

Full-field electroretinography (ERG) was performed using Espion Visual Electrophysiology System (Diagnosys Vision Ltd, Dublin, Ireland), and the International Society for Clinical Electrophysiology of Vision (ISCEV) standards in the proband. The protocol included rod-specific and standard bright flash ERG, both recorded after a minimum of 20-min dark adaptation. Following 10-min light adaptation, the photopic 30-Hz Flicker cone and transient cone ERG was recorded.

### Exome sequencing

Genomic DNA was extracted from peripheral blood. Whole exome sequencing was performed in both patients, using the following procedure: DNA was captured, and coding regions and splice sites were enriched using the capture kit Agilent SureSelect QXT Human All Exon V5. Sequencing was performed on an Illumina instrument following the manufacturer’s protocol. Targeted bioinformatic analysis of genes involved in Kabuki syndrome was done through locally developed pipelines, integrating BWA v0.7.10, Picard 1.80, GATK 3.50, ANNOVAR vSept2015. We selected variants from genes of interest, masking the rest of the data. The evaluation of variants used the following databases: dbSNPv142, ExAC 0.3, ClinVar 2016, HGMD 2016, LOVD, and the local database of variants. Prediction programs are as follows: SIFT, Polyphen2, MutationTaster, dbscSNV11, and HSF v.3.0. Variants were classified according to the recommendations of the American College of Medical Genetics [20]. Detected variants were validated and familial segregation performed by Sanger sequencing.

## Results

### Individual 1

**Fig. 1:**
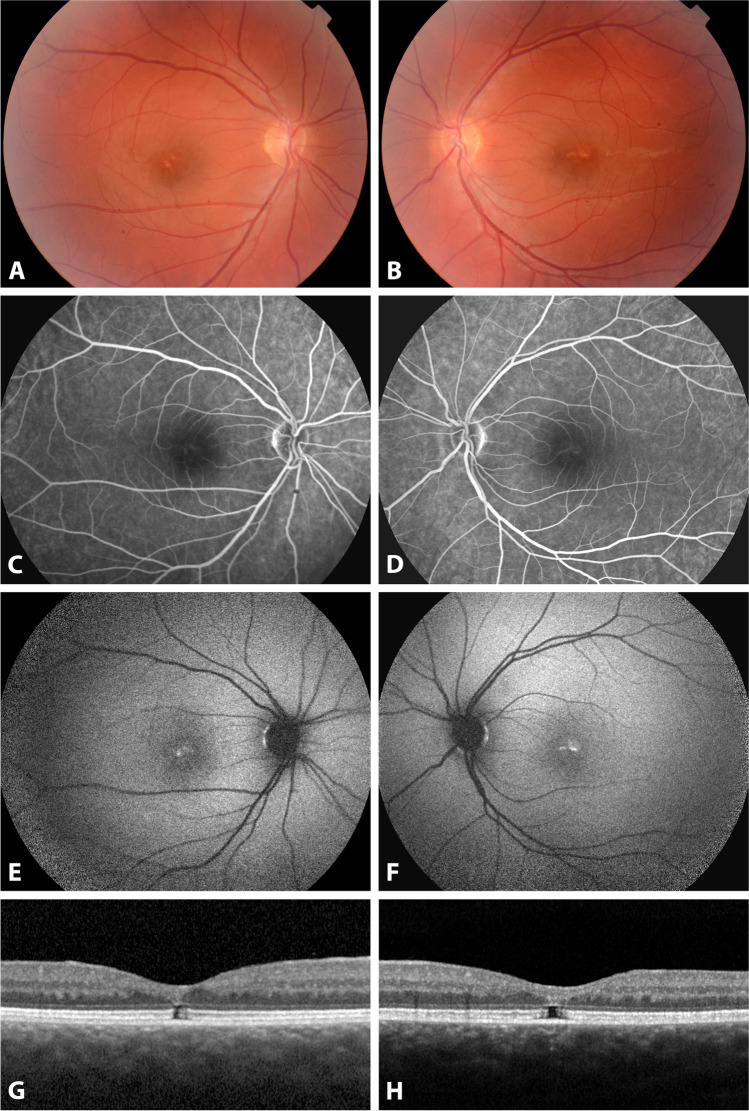
Fundoscopic color photo, fluorescein angiography, autofluorescence, and SD-OCT characteristics of patient 1, at age 22 years old, carrying a *de novo* c.14843C>G; p. (Ser 4948ter) novel variant in *KMT2D* gene (**A**–**H**)

A 22-year-old female came to our clinic to investigate reduced vision, noticed by herself and her work supervisors. Her Snellen best corrected visual acuity (BCVA) was 0.1 bilaterally. Fundus examination showed yellow pigmented deposits at the fovea of both eyes (Fig. 1A, B). Autofluorescence imaging (AF) showed hyperfluorescent material at the fovea (Fig. 1E, F), while fluorescein angiogram was within normal limits (Fig. 1C, D). Structural optical coherence tomography (SD-OCT) showed a bilateral gap at the photoreceptor layer with disruption of the ellipsoid layer and interdigitation layer, the external limiting membrane being intact (Fig. 1G, H). A full field ISCEV standard ERGs and VEP were within normal limits, while a kinetic Goldman visual field was constricted, with tubular appearance. The angio OCT was also within normal limits.

When the patient was 6 years old, she was sent for a genetic assessment and the clinical diagnosis of Kabuki syndrome was suspected at that time, following some typical features: moderate intellectual delay, clinodactyly, nail abnormalities, hypothyroidy, and hyperprolactinemia. A Marcus Gunn syndrome was noted as well as a bilateral ptosis. The patient, from Swiss origin, has a twin sister and two other siblings, all in good general health, as well as both unrelated parents. The genetic diagnosis was made when the patient was 23 years old and confirmed a heterozygote *de novo* c.14843C>G; p. (Ser4948ter) novel variant in *KMT2D* gene. Both parents were negative for the variant.

When she was between 10 and 12 years old, her then ophthalmologist found a Snellen BCVA of 0.9 in both eyes. However, the vision has been fluctuating in the right eye, requiring some occlusion treatment for suspected amblyopia.

Four years after the initial diagnosis of maculopathy, at age 26 years, the Snellen visual acuity improved to 0.3 BE, while the imaging OCT and AF remained stable, with no major changes. In addition, her visual field remained stable, and the tubular aspect was presumed to be due to an attentional deficit.

**Fig. 2:**
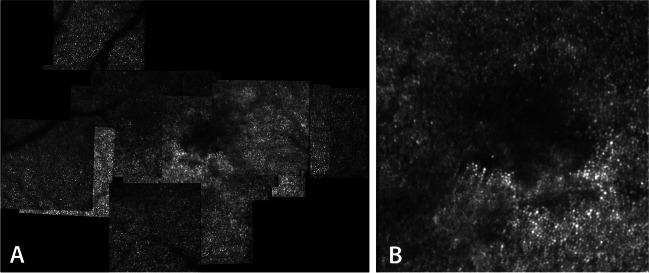
Adaptive optics right eye patient 1 showing a dark area on AO imaging, which is the location of suspected absent photoreceptors, or photoreceptors with changed structure

At that last visit at 26 years of age, she was working in our hospital in the attendance team. Adaptive optics showed a dark region, overlapping the focal disruption of EZ band at the fovea (Fig. 2A, B). The dark area on AO imaging is the location of suspected absent photoreceptors, or photoreceptors with changed structure, which does not provide an interface for waveguided incoming light.

### Individual 2

**Fig. 3:**
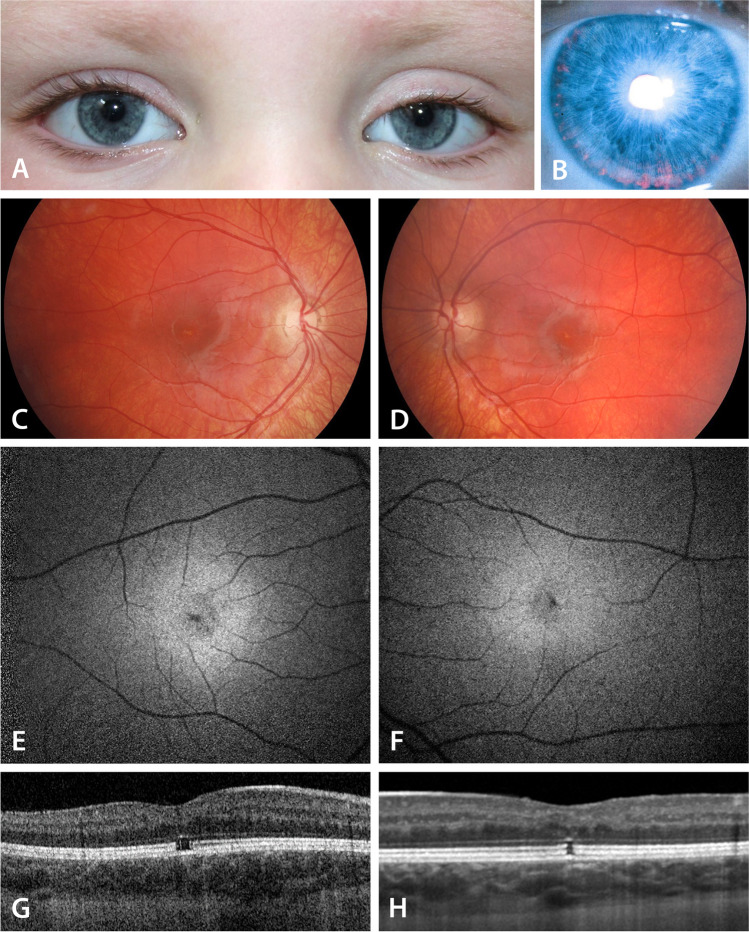
Facial features, iris transillumination (at age 4 years), fundoscopic color photo, autofluorescence, and SD-OCT characteristics of patient 2, with a *de novo* nonsense c.11119C>T; p. (Arg3707ter) pathogenic variant in *KMT2D* gene (**A–H**)

A 4-year-old girl born in France was diagnosed with Kabuki syndrome, following a developmental delay, cleft palate, as well as typical facial features including left eye ptosis (Fig. 3A). The Snellen visual acuity at that age was 0.2 in the right eye and 0.25 in the left. She had bilateral iris transillumination (Fig. 3B). The fundus examination showed white deposits at the fovea bilaterally (Fig. 3C, D). Autofluorescence imaging (AF) showed a hypofluorescent well delimited area at the fovea of both eyes, with very tiny hyperfluorescent dots around (Fig. 3C, D). SD-OCT showed a bilateral gap at the photoreceptor layer: defect at the level of ellipsoid zone and interdigitation zone (Fig. 3G, H). Full-field ISCEV standard ERGs were within normal limits.

Six years later at age 10, she started to complain of photophobia. Her Snellen visual acuity improved to 0.5 bilaterally, while the OCT findings did not change. Kinetic visual field was performed for the first time and was within normal limits.

Genetic testing identified a *de novo* nonsense heterozygote c.11119C>T; p. (Arg3707ter) pathogenic variant in *KMT2D* gene; both parents were negative for this mutation*.*

### Proteotype analysis suggests that both patients’ genotypes are pathogenic

**Fig. 4:**
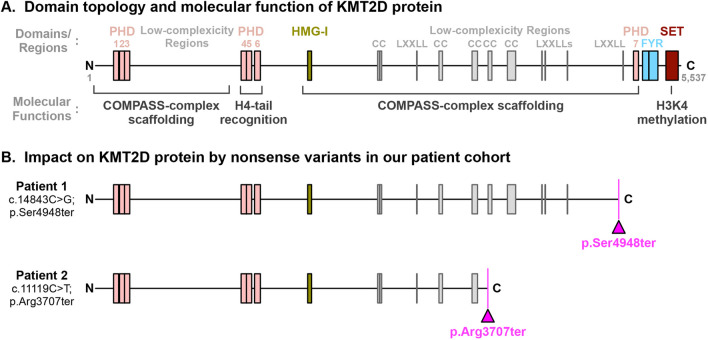
Proteotype analysis of both *KMTD2* de novo variants

The *KMT2D* gene encodes for the Histone-lysine N-methyltransferase 2D (KMT2D) protein, which is a member of type-2 histone H3 lysine 4 (H3K4) methyltransferase family that also includes Histone-Lysine N-methyltransferase 2C (KMT2C). The *KMT2D* gene co-localizes with lineage determining transcription factors existing on transcriptional enhancers, which play a key role in regulating development, differentiation, and metabolism. KMT2D is a protein consisting of 5537 amino acids that contains a catalytic SET domain—a tertiary protein structure with methylation function—at its C-terminus. It also possesses 7 plant homeostatic domains (PHDs) that recognize H4 histone tails, which allows for spatial guidance of the KMT2D catalytic SET domain to H3 histone tails (Fig. 4A) [21, 22]. In addition to its active enzymatic-role, KMT2D serves as a scaffold protein forming the core nuclear regulatory machinery known as the KMT2C/D COMPASS complexes (complex of proteins associating with Set1) [23]. In the complex, histone modification-associated proteins, such as WRAD (WDR5, RbBP5, ASH2L, and DPY30) and UTX, are hosted by the low-complexity regions and interaction motifs in KMT2D (e.g., high mobility group [HMG-I] domain and nuclear receptor interacting motifs [LXXLLs]) (Fig. 4A) [21, 23].

To assess the pathogenicity of the *de novo* nonsense variant in individual 1 (c.14843C>G; p. Ser4948ter), we analyzed its impact on KMT2D at the protein level. The early termination of *KMT2D* transcript is very likely to be susceptible to nonsense-mediated decay, resulting in haploinsufficiency. Assuming that there is a possibility for *KMT2D* mutant mRNA to avoid nonsense-mediated decay, the translated protein would lack the C-terminal PHD7 domain, FY-rich (FYR) domains, and catalytic SET domain present in normal KMT2D (Fig. 4B; upper panel). Thus, the mutant KMT2D will be enzymatically inactive and have limited scaffolding capability to form COMPASS complexes. Experimentally, the removal of the SET domain from the KMT2D protein, which is equivalent to the proteotypic impact of our patient’s genetic variant, significantly disturbs its protein stability in cells [24]. With these defects, the patient’s KMT2D mutant protein is not only enzymatically inactive, but also may be absent in cells due to nonsense-mediated mRNA-decay and protein degradation.

Compared to the individual 1’s KMT2D mutation, the pathogenic *de novo* nonsense variant in the second individual (c.11119C>T; p. Arg3707ter) resulted in a protein that is approximately 1200 amino acids shorter. This includes the loss of the previously listed domains and additional low-complexity regions, which serve as the binding platform for histone modification-associated proteins (Fig. 4B; lower panel). The proteotypic consequence by this nonsense variant is the same as that exhibited in individual 1 (i.e., enzymatically inactive, and prone to mRNA-decay/protein degradation), since it also results KMT2D mutant proteins without their expected C-terminal, PHD7, FYR domains, and SET domain. Thus, it is expected that the pathological impact of these two variants is to be the same, and the variability of clinical outcomes in these Kabuki syndrome patients may depend on non-*KMT2D* factors. This proteotypic finding could be applicable for more than 548 nonsense and frameshift variants of *KMT2D* gene without SET domain as reported in ClinVar and for similar future *KMT2D* gene variants.

## Discussion

There are only three reports describing patients with clinical Kabuki syndrome and documented lesions at the fovea. All three published cases did not have genetic confirmation, since the gene had not been known at that time. The first case was a 6-year-old boy with yellow-white deposits at the fovea, bilaterally. His VA was 0.5 but later improved to 1.0; full-field ERGs were within normal limits [18]. Few years later, a 21-year-old man was also reported with irregular foveal pigmentation and prepapillary gliosis. He described VA was 1.0 [25]. In 2011, one case of macular dystrophy with reduced VA of 0.25 in the right and 0.15 in the left was reported. The pattern ERG was abnormal, as well as OCT, showing irregular inner outer segment junction at the fovea [19].

Here, we report two additional cases of macular dystrophy in two unrelated patients with Kabuki syndrome 1 and *de novo* nonsense variants in *KMT2D*, one already described and the other one novel.

The common sign for all patients with macular lesions is yellow white deposits at the fovea of both eyes. These deposits are hyperautofluorescent on AF imaging in the first patient, while a delimited area of hypofluorescence was predominant in the second patient, corresponding more to the gap in the photoreceptor layer. An optical gap on OCT indicates cones are affected [26]. We can hypothesize that some differentiation abnormality at the fovea explains that visual function is reduced, but the exact extent is difficult to assess since VA is fluctuating, possibly to attentional deficit that patients with KS suffer from.

The attentional deficit also explains the tubular visual field, as the behavior, speed of walking and movement of our 26 years old patient did not correspond to a true tubular visual field. In addition, due to the young age of individual 2, and following a discussion with family members, any behavioral problems (such as sun gazing) were unlikely as explanation for the macular changes.

*KMT2D* encodes a large protein (593 kDa) a lysine specific methyltransferase 2D, that catalyze the mono-, di- and trimethylation of the lysine 4 on histone 3 [27]. Histone methylation is an epigenetic methylation by which expression of distinct genes and pathways are regulated at precise developmental stages. It is well established that in retinal development all six types of neurons and glial cells (photoreceptor, horizontal cells, bipolar cells, amacrine cells, ganglion cells, Muller Glial cells) are generated from common progenitor multipotent cells [27]. Recent study in a mouse model confirmed that histone methyltransferase activity in retinal progenitor cells, but not photoreceptors cells, is essential for normal differentiation and survival of the retina [28]. Following those findings, we can hypothesize some differentiation abnormality at the fovea explains the yellow white deposits. According to the previous study, the pathology is not at the photoreceptor level, but rather at a Muller cell level, regarding the aspect on OCT. This abnormality very likely happened at a very early age. Indeed, both our patients remain stable in terms of the retinal findings.

Most variants in KS are nonsense or frameshift mutations, likely resulting in mRNA degradation and contributing to protein haploinsufficiency [29]. The *KMT2D* haploinsufficiency has been suggested as being one of mechanisms underlying the pathogenesis of the disease. *KMT2D* may be directly or indirectly involved in the development and differentiation of the inner and outer segments of the foveal photoreceptors, in view of the irregular foveal EZ.

In summary, we present two additional unrelated patients with macular dystrophy and genetically confirmed Kabuki syndrome 1 where the abnormality of retinal differentiation likely happened at an early age.
